# *Mycobacterium tuberculosis*‐induced multiple tenosynovial masses with rice bodies: A case report

**DOI:** 10.1002/ccr3.8228

**Published:** 2023-12-19

**Authors:** Seyed Arman Moein, Reza Fereidooni, Reza Niakan, Aliasghar Kousari

**Affiliations:** ^1^ Bone and Joint Diseases Research Center Shiraz University of Medical Sciences Shiraz Iran; ^2^ Research Center for Non‐communicable Diseases Jahrom University of Medical Sciences Jahrom Iran; ^3^ Health Policy Research Center, Institute of Health Shiraz University of Medical Sciences Shiraz Iran; ^4^ Student Research Committee Shiraz University of Medical Sciences Shiraz Iran

**Keywords:** extrapulmonary tuberculosis, *Mycobacterium tuberculosis*, rice body, tenosynovitis, Xpert MTB/RIF assay

## Abstract

**Key clinical message:**

Tenosynovitis with rice bodies is a rare presentation, diagnosable by MRI. Surgical excision is the preferred treatment for tendon sheath masses containing rice bodies. The etiology was *Mycobacterium tuberculosis* in our case, highlighting the need to consider extrapulmonary TB in atypical presentations, ensuring effective treatment.

**Abstract:**

Rice body tenosynovitis is a rare clinical phenomenon with various etiologies. Inflammatory diseases such as rheumatoid arthritis and tuberculosis have been shown to be associated with this condition. Herein we report a 46‐year‐old male who presented with multiple masses of the dorsal and volar aspects of the left wrist. The masses did not cause significant pain or limitation of motion. Magnetic resonance imaging showed the presence of numerous hypointense bodies in the masses. During the surgical procedure, separate cysts originating from the synovitis of both the extensor and flexor compartments were identified along with white rice bodies within them. Masses were excised and Xpert MTB/RIF assay on the rice bodies was conclusive of *M. tuberculosis* (TB). The patient was discharged on anti‐TB medications with no complication or recurrence after 1 year of follow‐up.

## Graphical abstract

Masses containing rice bodies, originating from dorsal and volar tendon sheaths of the wrist.

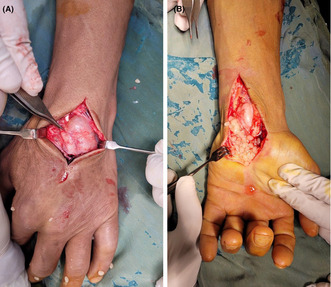

## 1 INTRODUCTION

First described in 1895, rice bodies are intrasynovial masses resembling rice grains.^1^ The exact etiology of these intrasynovial masses has not been established. Synovial microinfarction followed by synovial shedding and further encasement by fibrin has been implicated in rice body formation.^2^

In the literature, rice bodies have usually been observed in association with rheumatoid arthritis (RA), juvenile arthritis, osteoarthritis, or idiopathic causes.^3^, ^4^, ^5^ Rice bodies have also been reported in tendon sheath synovitis due to *Mycobacterium tuberculosis* (TB) infection.^6^, ^7^

Magnetic resonance imaging (MRI) is considered the preferred modality for diagnosing rice bodies and is essential prior to surgical excision to exclude other pathologies.^8^ Furthermore, due to possible mass effect, delayed management of rice bodies can cause neurovasculature compression^9^ or even result in osteoporosis of adjacent bones.^6^, ^7^

Here, we present a case of chronic tenosynovitis with multiple tenosynovial masses with rice bodies in the forearm, affecting both flexor and extensor tendon sheaths in a man with no history of RA to highlight the rare occurrence of rice body formation in musculoskeletal TB.

## 2 CASE PRESENTATION

A 46‐year‐old male, dairy farm worker, was referred to our clinic with multiple painless soft tissue masses of the left wrist since 1 year prior. The patient had no past medical or medication history. He denied any history of trauma or injury to his hand. There was no remarkable history of infection or inflammatory process. He denied the history of classic TB symptoms such as fever, weight loss, fatigue, cough, or night sweats. The contact history including living and occupational conditions was insignificant.

On physical examination, large mobile masses with minimal tenderness were noted on the dorsal and volar aspects of the wrist. No overlying warmth or erythema was observed. The patient had a normal range of motion in his fingers and wrist despite having mild discomfort. The Tinel sign was negative. Sensory and motor examination of the neurological components of the upper extremity was not remarkable.

Laboratory evaluation results revealed the patient to have normal erythrocyte sedimentation rate (ESR), negative C‐reactive protein (CRP), and normal white blood cell (WBC) count. Rheumatologic panel also turned out unremarkable.

**FIGURE 1:**
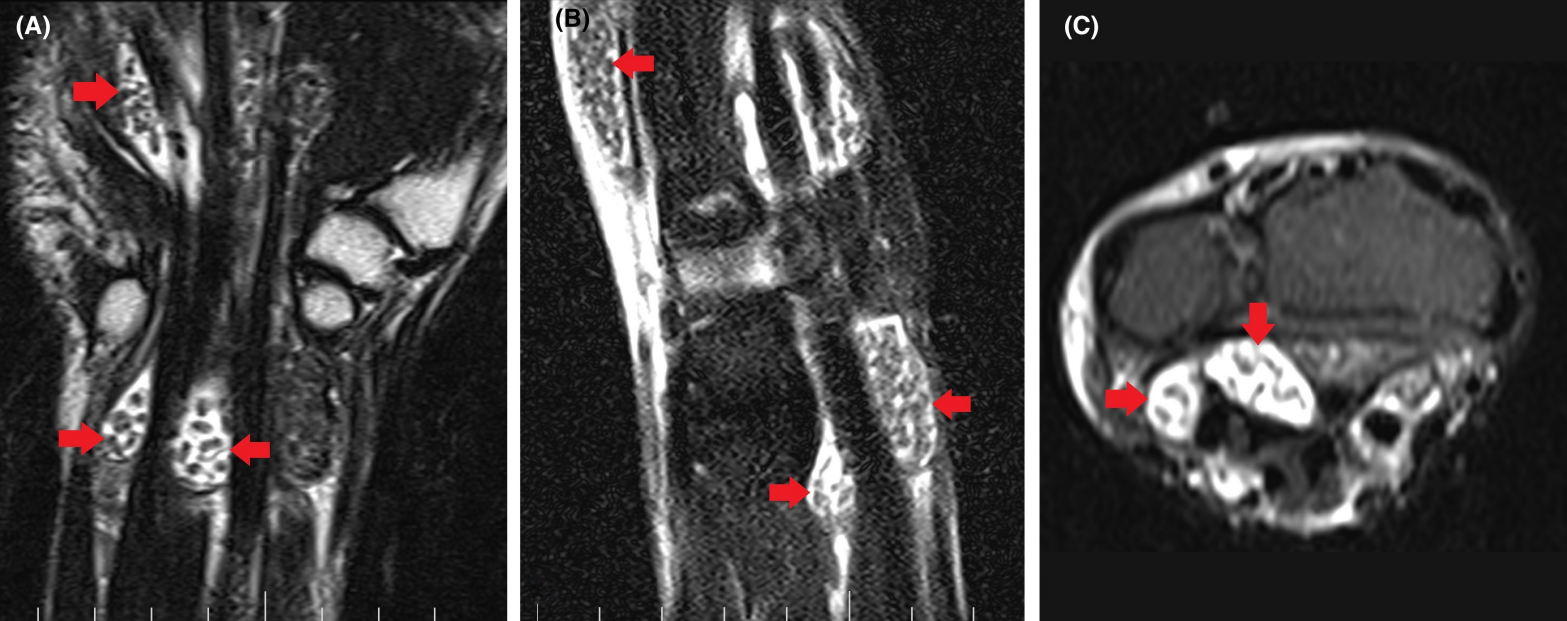
T2 sequences of the left wrist MRI; (A) coronal view, (B) sagittal view, (C) axial view.

Multiple lymph nodes up to 27 × 14 mm with abnormal appearance were seen on soft tissue ultrasonography of the left axilla. MRI of the right hand and wrist demonstrated multiple hypersignal masses in the flexor and extensor sheaths of the left wrist. The masses contained innumerable small round hypo‐intense bodies (Figure 1).

**FIGURE 2:**
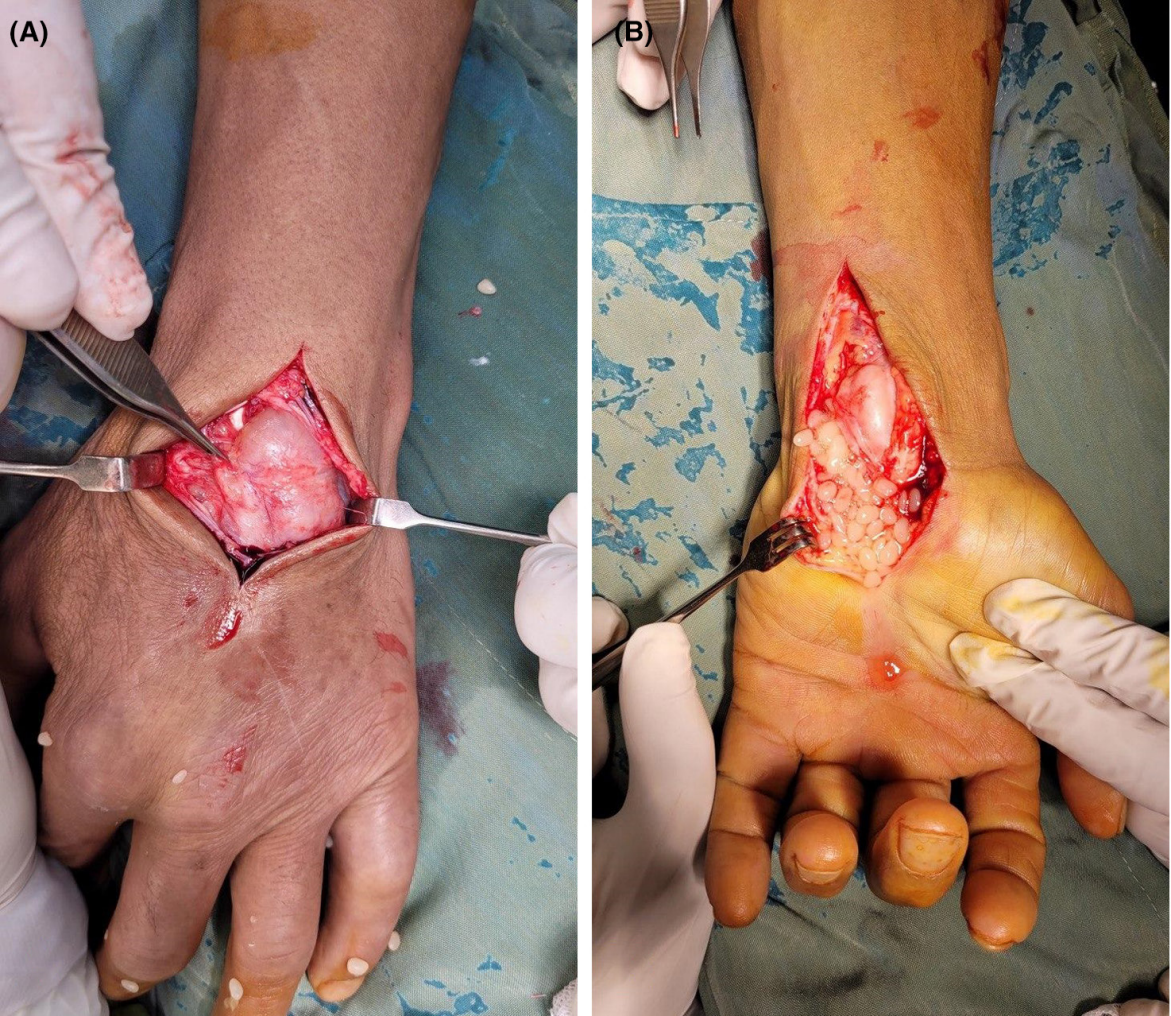
Intraoperative images; (A) rice bodies spilling out of the volar cysts, (B) unopened dorsal mass.

Considering the size and number of these masses and the patient's discomfort, excisional biopsy was planned. In surgery, through a curved linear incision on the volar aspect of the left wrist median nerve was explored proximally, and distally, the carpal tunnel was released. Masses were arising from the tendon sheaths synovium as expected so total flexor synovectomy was performed. Multiple synovial cysts were excised from the flexor compartment containing shiny white rice bodies which spilled out of the cysts (Figure 2A). Furthermore, through dorsal approach of the wrist, total synovectomy of extensor tendon sheaths was performed and the synovial masses were removed (Figure 2B).

Samples of rice bodies and tenosynovium were taken and sent to microbiology and pathology. Tissue culture turned negative. However, histochemical staining for acid‐fast bacilli demonstrated caseating necrosis with granulomatous inflammation but they were negative for microorganisms. Nucleic acid amplification with the Xpert MTB/RIF assay using the GeneXpert kit in the sample of rice bodies detected the presence of *M. tuberculosis* with no rifampin resistance. Acid‐fast stains of the sputum were negative and the diagnosis of extrapulmonary TB was made.

The patient was then referred to the infectious clinic for further management and was prescribed a 6‐month anti‐TB regime and vitamin B6.

## 3 DISCUSSION

Herein we presented a case of tenosynovitis with rice bodies associated with TB. In terms of TB incidence, Iran used to have a moderate TB incidence; however, with a decreasing trend it has reached the incidence of under 10 per 100,000 since 2020.^10^ Hence despite not being considered endemic, TB cases are not scarce either. Our patient stated no obvious risk factors of TB but contact history cannot be ruled out. Occupational contact with animals is also unlikely to be the cause of transmission, since usually the TB transmission route is human to livestock, and animals are accidental hosts.^11^

The pathogenesis of the rice bodies is still a matter of debate. They are thought to be secondary to chronic inflammation.^12^ They primarily consist of fibrin and collagen.^8^ Cheung et al.^2^, ^13^ found a protein composition comparable to synovium and Berg et al.^13^ observations revealed microvasculature in some rice bodies. While these studies are indicative of previous connections to the synovial membrane, other findings describe extra‐articular rice bodies suggestive of non‐synovial origin.^14^, ^15^ These fibrinous structures have been associated with chronic inflammatory diseases namely seropositive and seronegative RA, osteoarthritis, and juvenile arthritis.^4^, ^5^, ^15^ Rice bodies have also been documented instances in extrapulmonary TB,^3^, ^6^, ^7^ however these bodies in rheumatic diseases were slightly smaller.^16^ Histologic analysis of these loose bodies has shown caseating granulomas with *M. tuberculosis* and Langhans giant cells.^17^

Rice body synovitis affecting both wrist flexor and extensor tendons' compartments is an uncommon phenomenon. The development of rice bodies is not influenced by laterality, but many scholars believe that the dominant hand is more prone to be involved in TB.^16^, ^18^ Considering the association of TB infection and rice body formation and since the patient was left‐handed, laterality becomes a relevant consideration in our case.

Late presentation is often encountered since some patients only experience mild pain, and a little aching or stiffness.^16^ Delayed diagnosis is a matter of concern, especially when rice bodies are accompanied by TB, as infection can lead to osteoporosis of adjacent bones.^6^, ^7^ Tendon rupture, loss of range of motion, and nerve compression with subsequent carpal tunnel syndrome are other repercussions.^6^, ^9^, ^19^ In the cases of TB, prompt diagnosis is crucial to promptly initiate antituberculous medication, thereby preventing further complications.

Rice bodies can be visualized via MRI and ultrasonography. On ultrasonography, they are represented by hypoechoic nodules but it is very challenging to differentiate them from synovial chondromatosis.^8^ On T1‐ and T2‐weighted MRI images, they appear as hypo‐intense nodules in synovial fluid, easily distinguishable from synovial chondromatosis which demonstrates hyperintensity appearance on T2‐weighted images.^8^ Given the rarity of rice bodies, they may not initially be considered in the differential diagnosis by the physician. In such cases, the use of medical imaging becomes necessary. However, when rice bodies are visualized through imaging, a patient's history of tuberculosis (TB) and rheumatic diseases can be quite helpful in determining the underlying cause. The diagnosis of extrapulmonary TB can be approached through various methods, with varying levels of accuracy.^20^ For instance, a negative result on the IFN‐γ releasing assay (IGRA) conducted on a blood sample can be valuable in excluding extrapulmonary TB. Additionally, the Xpert assay exhibits relatively high sensitivity and specificity in detecting extrapulmonary TB and is recommended for non‐respiratory specimens.^20^ Diagnosis of RA, other inflammatory arthropathies, and trauma‐associated rice bodies are based on relevant patient history and laboratory examinations.

Clinical management of rice bodies varies in the literature and there is a paucity of information regarding the best possible treatment. Conservative management has not been suggested in the literature.^12^, ^21^ There have been reports of rice body removal through aspiration when the mass is symptomatic.^4^ According to Popert et al.^3^ lavage and aspiration have shown great efficacy in treating rice bodies. This therapeutic approach seems to be viable in larger joints like knees and shoulders, where rice bodies are more commonly observed. however, execution of this method in smaller joints like wrists can be challenging.^18^ Moreover, in our case, due to multiple separated synovial masses, aspiration was not feasible.

For mycobacterial infections, surgical debridement and tenosynovectomy accompanied by an antituberculous regime are required to prevent any further recurrence of the disease.^6^, ^16^ Mass removal through surgical means often yields favorable outcomes in terms of wrist function recovery, symptom alleviation, and recurrence rate.^12^, ^18^, ^22^

The recurrence of rice bodies has been reported in the literature. In RA patients, rice body recurrence is rarer and has been reported once in a patient with bilateral flexor synovitis.^5^ There have been some cases with delayed diagnosis or misdiagnosis of TB, leading the patient to undergo secondary surgeries.^7^, ^16^ The recurrence rate was up to 60% in some studies where surgery was performed without chemotherapy for TB.^16^ In that light, the administration of anti‐TB chemotherapy is necessary to effectively treat TB infection and prevent the reoccurrence of rice bodies.

Finally, our case was characterized by progressively enlarging masses over the dorsal and volar aspects of the wrist in a patient with no past medical record. Rice body synovitis of separate compartments of the wrist is a rare phenomenon. In conclusion, we believe that surgeons should consider various etiologies while encountering rice bodies in practice. Before surgery history, examination and diagnostic workup for rheumatic disease should be done. Physicians should also look for the presence of TB in other sites, especially pulmonary TB. After surgery, diagnostic tests including staining and nucleic acid amplification can help detect mycobacterium in the tissue. Multidisciplinary management of surgery in addition to medical treatment is necessary in such cases. Fortunately, our patient was followed up 1 year after surgery and showed no signs of recurrence or complication.

### 3.1 Relevant TB protection in healthcare setting

As per Guidelines for Preventing the Transmission of *M. tuberculosis* in Health‐Care Settings recommended by the Center for Disease Control and Prevention (CDC),^23^ individuals with extrapulmonary TB are typically not contagious unless they have coexisting pulmonary TB, nonpulmonary TB affecting the oral cavity or larynx, or extrapulmonary TB with an open, highly concentrated abscess or lesion. Also, there's an increased risk of contagion when extensive drainage from the abscess or aerosolization of the drainage fluid occurs. Irrigating TB abscesses is also considered an aerosol‐generating procedure.^23^

In our patient, due to our uncertainty regarding the potential association of rice bodies with TB, we did not assess the patient's pulmonary TB status preoperatively and a conventional acid‐fast stain on the sputum smears was performed postoperatively. Serving as a valuable lesson, this highlights the even higher importance of maintaining TB precautions, when operating suspicious masses or abscesses in patients whose pulmonary TB status is undetermined.

In the context of the presence of *M. tuberculosis* in the tenosynovial mass with rice bodies, these masses however not abscesses, and even though they contain minimal fluid, could be considered lesions where “the concentration of the organism is high”. This presents an intriguing scenario where there's a potential risk of contagion. While our patient did not have pulmonary TB—suggested by three negative sputum smears, the presence of *M. tuberculosis* within these masses, and the surgical excision of these masses, may have created conditions that warrant attention to TB infection control. Furthermore, the risk of contagion or lack thereof in patients with initially negative sputum smear is a matter of debate.^24^ The Xpert MTB/RIF assay is also the leading consensus for determining the need for isolation based on sputum testing.^24^

Accordingly, surgeons and health care personnel are recommended to don proper personal protection equipment, when opening or draining lesions containing confirmed or suspected *M. tuberculosis*, regardless of patient's pulmonary involvement. Respiratory protection against TB for healthcare staff and individuals visiting infectious TB patients includes N95 respirators that meet or exceed the filtration efficiency standard of 95%, as specified by the CDC and the National Institute for Occupational Safety and Health (NIOSH). The FFP2 respirator, which has a filtration efficiency of at least 92% is certified by European Conformity (CE). The World Health Organization (WHO) endorses either of these standard.^24^

## AUTHOR CONTRIBUTIONS

**Seyed Arman Moein:** Data curation; investigation; writing – original draft. **Reza Fereidooni:** Validation; visualization; writing – review and editing. **Reza Niakan:** Investigation; writing – original draft. **Aliasghar Kousari:** Conceptualization; data curation; investigation; project administration; resources.

## FUNDING INFORMATION

Not applicable.

## CONFLICT OF INTEREST STATEMENT

The authors declare no conflict of interest in this study.

## CONSENT

Written informed consent was obtained from the patient to publish this report in accordance with the journal's patient consent policy.

## Data Availability

The data that support the findings of this study are available on reasonable request from the corresponding author. The data are not publicly available due to privacy or ethical restrictions.
